# Comparative Analysis of the Chloroplast Genomes of *Quercus × morehus* and the Presumptive Parents *Q. wislizeni* and *Q. kelloggii* (Fagaceae) from California

**DOI:** 10.1128/mra.00321-22

**Published:** 2022-06-13

**Authors:** Alejandro Garcia, Althea C. Katada, Alyssa Serrano, Adrea Gonzalez-Karlsson, Angel Carrillo, Angelica Castellanos, Azucena Mendez-Gomez, Carlos J. Flores, Christopher Limon, Cynthia Lopez, Daniela Rosas-Uribe, Dylan J. Hidalgo, Ephraim C. Melgarejo, Erica L. Estamo, Faith Mora, Gabino Guzman, Jason F. Morones, Jeffery R. Hughey, Jennifer Sanchez-Mendoza, Jimena M. Parra, Joaquin Perez, Joe H. Perez, Joel Viorato Arambula, Juan S. Chavez, Juan R. Figueroa, Juan Rodriguez, Kevin Cardenas, Leslie Trejo, Lizbeth D. Lozano-Ruiz, Loreli Gonzalez, Lorena L. Vargas, Marc Anthony Trujillo, Mariana Rangel, Martin R. Delgado, Mayra A. Ibarra-Moreno, Nancy Chitica Villalobos, Priscila Corona, Quinn Snowden, Roberto Vargas, Robin B. Staretorp, Stephanie Martin, Victor M. Zavala

**Affiliations:** a Division of Mathematics, Science, and Engineering, Hartnell College, Salinas, California, USA; University of California, Riverside

## Abstract

Here, we present the complete chloroplast genomes of *Quercus × morehus*, *Q. wislizeni*, and *Q. kelloggii* from California. The genomes are 161,119 to 161,130 bp and encode 132 genes. *Quercus × morehus* and *Q. wislizeni* are identical in sequence but differ from *Q. kelloggii* by three indels and eight SNPs.

## ANNOUNCEMENT

*Quercus morehus* Kellogg, Abram’s oak, was originally proposed from a single specimen from near Clear Lake, CA (1). It was described as a small tree (9.14 m) with black bark, oblong-lanceolate leaves, and oblong nuts. Greene (2) was the first to study *Q. morehus* and concluded it was a hybrid between the interior live oak *Q. wislizeni* A. DC. and the black oak *Q. kelloggii* Newb. Subsequent authors agreed with this hypothesis, including Jepson who itemized six observations supporting the hybrid conclusion (3–7). Many oak chloroplast genomes have been sequenced to date (8–10); however, the genomes of *Quercus × morehus*, *Q. wislizeni*, and *Q. kelloggii* have not been analyzed. To contribute to the bioinformatics of *Quercus × morehus* and these closely related *Quercus* species, we assembled and characterized the complete chloroplast genomes of the presumptive hybrid and parents.

The leaves of three adjacent specimens were collected in Groveland, California (37°51'22.2"N 120°13′36.9"W) and deposited at Hartnell College under voucher numbers HCC 268 to 270. The DNA was extracted using the DNeasy Blood and Tissue kit (Qiagen) following two modifications: the binding step was centrifuged at 4,000 *g* for 3 min and the DNA was eluted after incubation for 7 min in 40 μL TAE (11). The 150 bp PE library was constructed with the NEBNext Ultra II DNA Library Prep kit (New England BioLabs) and sequenced by Novogene on the Illumina NovaSeq 6000. The analysis yielded 40,590,890 (*Quercus × morehus*), 17,672,202 (*Q. wislizeni*), and 14,854,920 (*Q. kelloggii*) reads. The adapters and low quality reads were removed using the Trim Adapters and Trim Low Quality default settings with the BBDuk plugin in Geneious Prime 2019.1.3 (Biomatters Limited). The genomes were assembled by mapping reads onto the reference sequence of *Q. agrifolia* Née var. *agrifolia*, GenBank accession number OK634019 (12) using the Medium Sensitivity/Fast setting in Geneious Prime. The mapping coverage for *Quercus × morehus* was 4,323×, *Q. wislizeni* 1,547×, and *Q. kelloggii* 1,885×. The gaps were closed by iterative mapping using the same settings in Geneious Prime. The annotation was performed using the default settings in GeSeq (13), followed by manual adjustments according to NCBI ORFfinder and Sequin 15.5 (14).

**FIG 1:**
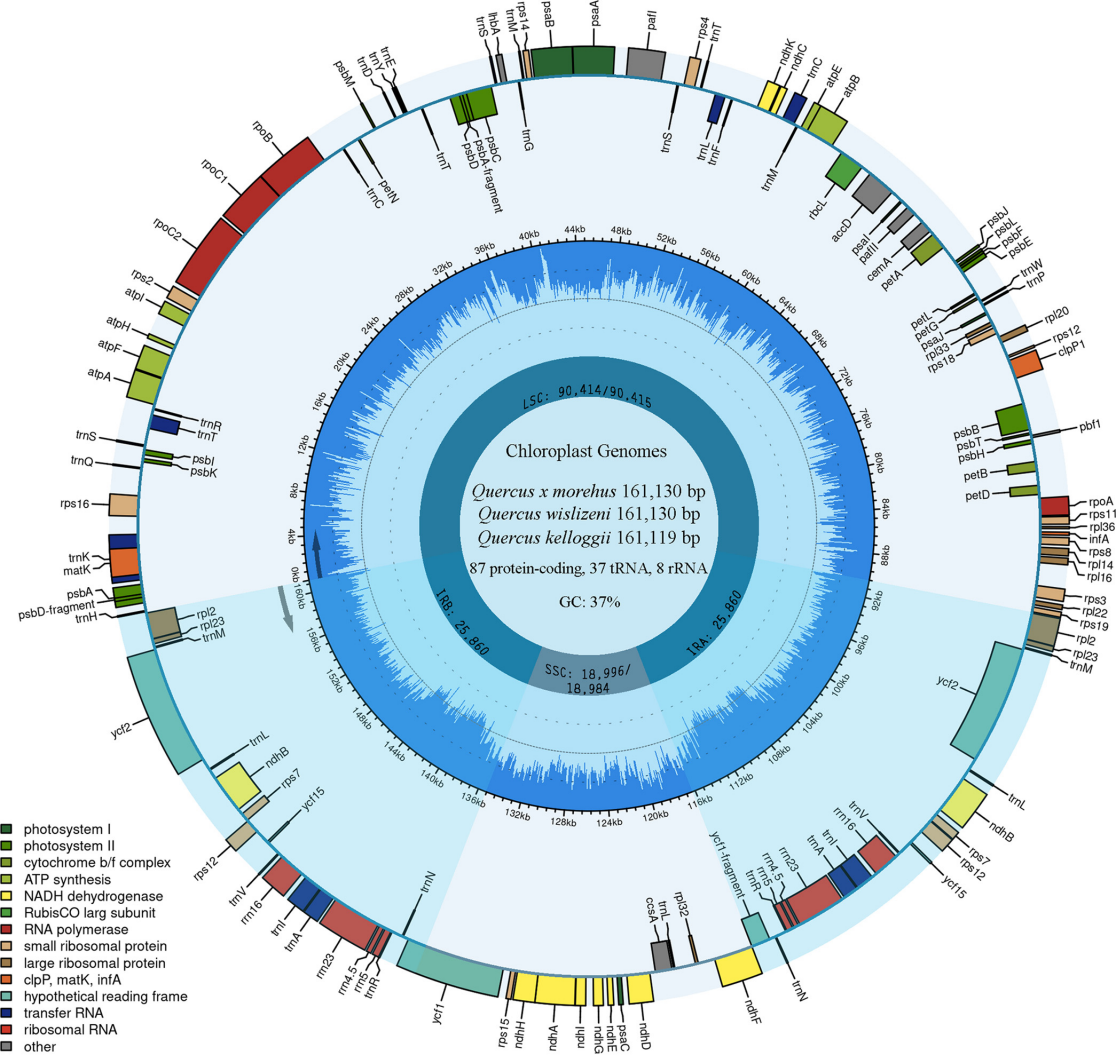
Complete chloroplast genomes of *Quercus × morehus*, *Q. wislizeni*, and *Q. kelloggii*. The genomes were annotated using GeSeq (13), NCBI ORFfinder and Sequin 15.5 (14), and mapped with CHLOROPLOT (17). The innermost ring identifies the LSC, SSC, and the two inverted repeats. The numbers before the forward slash correspond to *Quercus × morehus* and *Q. wislizeni*, and the numbers after the slash represent *Q. kelloggii*. The next ring displays the GC content and direction of transcription, as indicated by the two arrows. The final ring shows the genes. Genes transcribed clockwise are on the inside, while counterclockwise transcriptions are on the outside the circle. The color coding corresponds to genes of different groups as listed in the key in the bottom left.

The complete chloroplast genomes of *Quercus × morehus*, *Q. wislizeni*, and *Q. kelloggii* were 161,130, 161,130, and 161,119 bp in length, respectively, and displayed the characteristic flowering plant quadripartite structure (15). Gene content and organization of the three genomes are identical to other oaks classified in section Lobatae (8, 10, 12, 16). The three genomes showed a GC content of 37.0% and contained 132 genes, including 87 protein-coding, 37 tRNA, 8 rRNA genes (Fig. 1). The chloroplast genomes of *Quercus × morehus* and *Q. wislizeni* were identical in sequence but differed from *Q. kelloggii* by three indels and eight SNPs (five were located in noncoding and three in coding regions). Two of the three coding mutations were silent; however, the third altered the stop codon of the *ndhF* gene by 18 bp in *Quercus × morehus* and *Q. wislizeni*.

### Data availability.

The complete chloroplast genome sequences of *Quercus × morehus*, *Q. wislizeni*, and *Q. kelloggii* are available in GenBank under accession numbers OM541585, OM541583, and OM541584. The Illumina sequencing data for all three specimens are available under BioProject PRJNA818320. The reference genome for the annotation was *Q. agrifolia* var. *agrifolia* (GenBank accession number OK634019).
